# Induction of Efficacy Expectancies in an Ambulatory Smartphone-Based Digital Placebo Mental Health Intervention: Randomized Controlled Trial

**DOI:** 10.2196/20329

**Published:** 2021-02-17

**Authors:** Esther Stalujanis, Joel Neufeld, Martina Glaus Stalder, Angelo Belardi, Marion Tegethoff, Gunther Meinlschmidt

**Affiliations:** 1 Division of Clinical Psychology and Psychiatry Department of Psychology University of Basel Basel Switzerland; 2 Department of Clinical Psychology and Cognitive Behavioral Therapy International Psychoanalytic University Berlin Berlin Germany; 3 Division of Clinical Psychology and Epidemiology Department of Psychology University of Basel Basel Switzerland; 4 Institute of Psychology RWTH Aachen Aachen Germany; 5 Department of Psychosomatic Medicine University Hospital Basel and University of Basel Basel Switzerland

**Keywords:** digital placebo effect, efficacy expectancies, ecological momentary assessment, mHealth, mobile phone, placebo effect, randomized controlled trial, smartphone-based intervention

## Abstract

**Background:**

There is certain evidence on the efficacy of smartphone-based mental health interventions. However, the mechanisms of action remain unclear. Placebo effects contribute to the efficacy of face-to-face mental health interventions and may also be a potential mechanism of action in smartphone-based interventions.

**Objective:**

This study aimed to investigate whether different types of efficacy expectancies as potential factors underlying placebo effects could be successfully induced in a smartphone-based digital placebo mental health intervention, ostensibly targeting mood and stress.

**Methods:**

We conducted a randomized, controlled, single-blinded, superiority trial with a multi-arm parallel design. Participants underwent an Android smartphone-based digital placebo mental health intervention for 20 days. We induced prospective efficacy expectancies via initial instructions on the purpose of the intervention and retrospective efficacy expectancies via feedback on the success of the intervention at days 1, 4, 7, 10, and 13. A total of 132 healthy participants were randomized to a prospective expectancy–only condition (n=33), a retrospective expectancy–only condition (n=33), a combined expectancy condition (n=34), or a control condition (n=32). As the endpoint, we assessed changes in efficacy expectancies with the Credibility Expectancy Questionnaire, before the intervention and on days 1, 7, 14, and 20. For statistical analyses, we used a random effects model for the intention-to-treat sample, with intervention day as time variable and condition as two factors: prospective expectancy (yes vs no) and retrospective expectancy (yes vs no), allowed to vary over participant and intervention day.

**Results:**

Credibility (β=−1.63; 95% CI −2.37 to −0.89; *P*<.001) and expectancy (β=−0.77; 95% CI −1.49 to −0.05; *P*=.04) decreased across the intervention days. For credibility and expectancy, we found significant three-way interactions: intervention day×prospective expectancy×retrospective expectancy (credibility: β=2.05; 95% CI 0.60-3.50; *P*=.006; expectancy: β=1.55; 95% CI 0.14-2.95; *P*=.03), suggesting that efficacy expectancies decreased least in the combined expectancy condition and the control condition.

**Conclusions:**

To our knowledge, this is the first empirical study investigating whether efficacy expectancies can be successfully induced in a specifically designed placebo smartphone-based mental health intervention. Our findings may pave the way to diminish or exploit digital placebo effects and help to improve the efficacy of digital mental health interventions.

**Trial Registration:**

Clinicaltrials.gov NCT02365220; https://clinicaltrials.gov/ct2/show/NCT02365220.

## Introduction

### Background

Mental disorders are highly prevalent and cause a high global burden of disease [1,2]. A large proportion of persons with mental disorders do not receive adequate treatment, among other reasons, due to the limited availability of face-to-face psychotherapy, particularly in low- and middle-income countries [3]. To address these challenges, the World Health Organization has defined research priorities to improve the lives of people with mental disorders [4]. One of these research priorities is the development of mobile and information technologies to increase access to evidence-based care. Furthermore, several people with mental disorders do not respond to traditional face-to-face psychotherapy [5]. Therefore, new forms of treatment are required [6].

Numerous health-related smartphone apps have been applied, for instance, for the prevention and treatment of depressive or anxiety disorders [7-10]. Research on the efficacy and effectiveness of these apps is only at its beginning. Recent meta-analyses of randomized controlled trials (RCTs) reported small to moderate effect sizes, suggesting that delivering psychological treatment with smartphone-based devices may be an efficacious approach to treat, for instance, anxiety and depressive symptoms [11,12]. However, there is a lack of knowledge on the potential mechanisms of change in mental health interventions delivered by smartphone apps. Firth et al [11] found that effect sizes of smartphone interventions were smaller in studies with active control conditions, as compared with waitlist or inactive controls, suggesting that the use of a smartphone itself may provide psychological benefit. In this regard, Torous and Firth [13] considered the consequences of a potential placebo effect and introduced the concept of a digital placebo effect, defined as “placebo-like effects seen from mobile health interventions, such as smartphone apps.”

The placebo effect is understood as a range of positive changes occurring after patients have been provided with an inert or inactive treatment [14]. Traditionally, placebo effects have been discussed in the field of blinded RCTs in which study participants in a control group received placebos in the form of inert pills or sham procedures. Active treatments need to outperform placebos in RCTs to be considered effective. In this context, placebo effects should be minimized to ensure a valid investigation of the drug’s efficacy [15].

An important factor underlying placebo effects are outcome expectancies of patients, which means that a therapeutic intervention can produce a placebo effect because the person receiving the treatment believes it will have an effect [14,16]. Previous studies found positive associations between favorable outcome expectancies of patients and positive therapeutic effects for a wide range of medical conditions and mental disorders, such as Parkinson disease, hypertension, depression, anxiety, and pain [16-20]. Accordingly, some authors claimed that placebo effects should be maximized to improve treatment outcomes by enhancing patients’ expectancies [15,20]. Gruszka et al [21] stated that despite its therapeutic potential, the validation of efficacy of placebo interventions, such as expectancy interventions, has been neglected. Mobile apps provide a novel approach to provide highly standardized expectancy interventions in a blinded manner and have several advantages for investigating expectancy interventions [21].

In summary, there is an urgent need to (1) further scrutinize smartphone-based mental health interventions in the context of the World Health Organization grand challenge on the development of mobile and information technologies to increase access to evidence-based care; (2) explore potential mechanisms of change underlying smartphone-based mental health interventions that are already widespread but are not validated; and (3) scrutinize and exploit efficacy expectancies as a potential factor underlying placebo effects, using smartphone-based interventions with respect to their methodological advantages.

### Objectives

Previous studies in the context of digital placebo effects introduced the concept [13], focused on methodological recommendations for RCTs of smartphone-based interventions [21,22], or used a sham version of an active app as control condition [23]. However, to the best of our knowledge, no study has investigated efficacy expectancies as a potential mechanism of the digital placebo effect in a particularly designed inert smartphone-based mental health intervention. Therefore, the aim of our study is to investigate whether efficacy expectancies could be successfully induced in a smartphone-based placebo mental health intervention. We designed a smartphone-based placebo mental health intervention that lasted 20 consecutive days and induced different efficacy expectancies regarding the effects of the intervention on mood and stress in participants, with emotional state being associated with major depression and anxiety as the most frequent mental disorders [1,24]. We differentiated between *prospective expectancy*, which we induced at the beginning of the smartphone-based placebo mental health intervention, and *retrospective expectancy*, which we induced during several days, immediately after participants had completed the smartphone-based digital placebo mental health intervention. We hypothesized that trajectories of efficacy expectancies throughout the smartphone-based placebo mental health intervention differed between conditions. Furthermore, we hypothesized that efficacy expectancies were highest in the combined expectancy condition, followed by a comparable level in the prospective expectancy condition and the retrospective expectancy condition, and were lowest in the control condition.

## Methods

### Overall Study Procedure

We report the results of a randomized, controlled, single-blinded, superiority trial with a multi-arm parallel design, registered at Clinicaltrials.gov (Identifier: NCT02365220). The aim of this larger study is to investigate the placebo effect in a smartphone-based mental health intervention. The Institutional Review Board of the Department of Psychology of the University of Basel, Switzerland, approved the study protocol (no.: 005-14-2). All participants provided written informed consent in accordance with the Declaration of Helsinki. The study was conducted between February and October 2015 at the Department of Psychology of the University of Basel, Switzerland. The study consisted of an introductory session and 20 consecutive days of ambulatory smartphone-based intervention (Multimedia Appendix 1). The data presented here were collected at the introductory session and on intervention days 1, 7, 14, and 20 when efficacy expectancies had been measured with the Credibility and Expectancy Questionnaire (CEQ; for further details see the section *Outcome Variable: Efficacy Expectancies With CEQ*).

### Introductory Session

In the introductory session, we informed participants about the aim and procedure of the study and assessed inclusion criteria, sociodemographic information, and information on their general and mental health (Patient Health Questionnaire—German version [25,26] and Perceived Stress Scale—10-items version [27,28]). Irrespective of potential condition assignment, we informed all participants that in our study we would be interested in how mood and perceived stress fluctuated in daily life and whether smartphones would be suitable to assess their temporal trajectories. We instructed participants to download and install the *ohmage* app [29] from the Google Play Store on their Android-based smartphones. *Ohmage* is an open mobile system consisting of a smartphone app for self-reported data collection and a server system for web-based data storage, management, and administration. We set up and maintained our own *ohmage* server at the information technology division of the Department of Psychology.

### Smartphone-Based Digital Placebo Mental Health Intervention

The second part of the study consisted of a 20-day smartphone-based ambulatory mental health intervention. Participants started with the intervention 3 days after they had attended the introductory session. The detailed procedure of each session is illustrated in Multimedia Appendix 2. The placebo mental health intervention consisted of a green picture or a mock sound, delivered in a video file on the Enterprise Feedback Suite survey, which participants accessed via their Android-based smartphones. The videos lasted for 2 minutes each and alternated daily between green color and mock sound. Regarding the mock sound, we told participants in the initial instructions that the sound would be a very soft tone acoustically not perceivable for the human ear and completely innocuous. On intervention days 1, 4, 7, 10, and 13, we asked participants to take a self-portrait with their smartphone camera within the *ohmage* app. After this second self-portrait, we provided participants with written feedback regarding the self-portrait in the *ohmage* app that we had programmed in advance. On intervention days 1, 7, 14, and 20, we asked participants to rate efficacy expectancies, measured with the CEQ (for further details see the section *Outcome Variable: Efficacy Expectancies With CEQ*).

### Induction of Efficacy Expectancies

**Figure 1:**
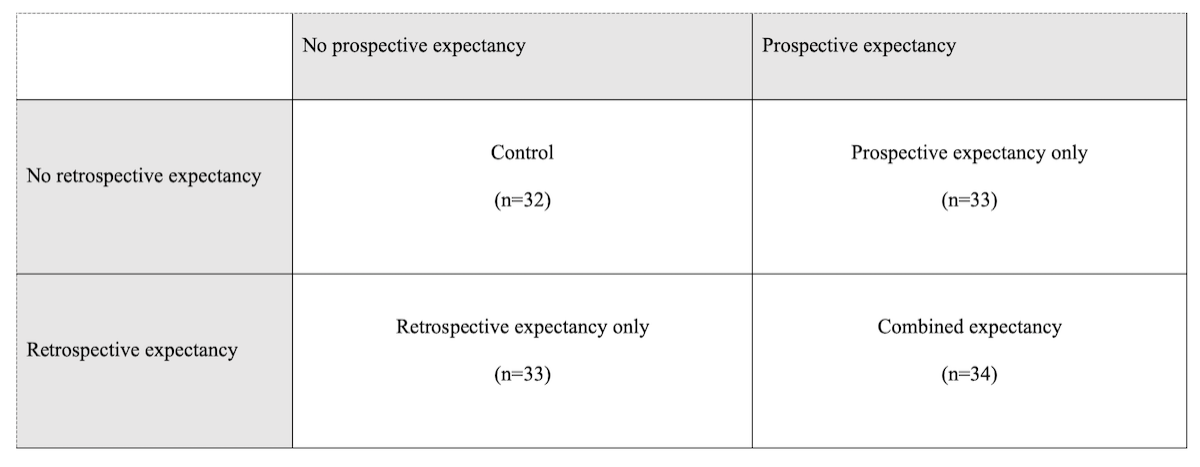
Fourfold table of different conditions.

Efficacy expectancies in the 4 conditions were induced in 2 ways: (1) instructions on the purpose of the study on intervention day 1 and (2) the written feedback following the second (ie, post placebo intervention) self-portrait on intervention days 1, 7, 14, and 20. The initial instructions in our experiment served to induce prospective expectancies in participants, the feedback on the self-portraits served to induce retrospective expectancies (see Figure 1 for a fourfold table of the 4 conditions). In the control condition, on intervention day 1, participants received the same information about the purpose of the study as in the introductory session, according to which, we were interested in establishing how mood and perceived stress fluctuated in daily life and whether smartphones were suitable to assess their temporal trajectories. We did not give any explanation on the purpose of the self-portraits. After having sent the post placebo intervention self-portrait, participants received a *Thank you* message from us. In the prospective expectancy–only condition, on intervention day 1, we told participants that we were interested in whether a smartphone-based intervention lasting several weeks might have a positive effect on mood and stress perception. Moreover, we explained that previous studies had demonstrated that green light and soft tones beyond the acoustic detection threshold had positively affected the activity of certain brain regions, such as the insular lobe. We provided further details on the role of the insular lobe in the formation of unpleasant emotions and the release of stress hormones. We told participants that we assumed that daily exposure to a green picture or an inaudible sound would positively affect their mood and perceived stress in general and their ratings of emotional pictures in particular. Regarding self-portraits, the procedures were identical to the control condition. In the retrospective expectancy–only condition, initial instructions on the purpose of the study were identical to the control condition. Regarding the self-portraits, we told participants that the *ohmage* app would compare the emotional facial expression of the 2 self-portraits, which might differ according to the levels of mood and perceived stress. After having taken the post placebo intervention self-portrait, participants were informed by the *ohmage* app that their picture was currently being analyzed. Then, we provided participants with feedback that their stress level and mood had improved to a certain extent. We had programmed the reported levels of improvement for mood and perceived stress in advance. They were identical for each participant but different for each self-portraying intervention day, to make the deception more plausible. In the combined expectancy condition, initial instructions on the purpose of the study were identical to the prospective expectancy–only condition. Procedures regarding the analysis of the self-portraits were identical to the retrospective expectancy–only condition (for detailed instructions, see Multimedia Appendix 3).

### Outcome Variable: Efficacy Expectancies With CEQ

We measured efficacy expectancies as outcome variables with CEQ, which was developed to measure treatment expectancy and rationale credibility in clinical outcome studies [30]. Further details on the structure of the CEQ and how we built the subscales’ credibility and expectancy can be found in the study by Smeets et al [31]. The CEQ exhibits good psychometric properties [30]. It was administered at the introductory session (day 0) and on intervention days 1, 7, 14, and 20, after participants had completed the smartphone-based mental health intervention and the International Affective Picture System picture ratings.

### Participants

Participants were recruited from the bachelor student body of the University of Basel, Switzerland, in psychology and other lectures, where we presented our study. Advertisements of our study were posted on the website of the psychology students’ Facebook group and on local bulletin boards of the Department of Psychology. We compensated psychology students with signatures for study completion, which they required as parts of their bachelor’s studies. If participants dropped out before completion, compensation was granted proportionally. For students of other faculties, we offered participation in a lottery drawing for a tablet computer as compensation. For study participation, students had to use their own smartphones for 20 consecutive days. Only participants with access to an Android-based smartphone were included because the app we used in our study was available for Android only, that is, potentially interested participants with iOS-based smartphones could not be included in the study. Due to low recruitment rates during the initial data collection, students with access to an Android-based tablet computer were also accepted for participation in our study (7 participants in total). The following inclusion criteria were applied: no severe visual impairment, no dyschromatopsia, no severe defective hearing, no regular intake of medication (eg, antidepressants), and no severe mental disorders (eg, schizophrenia, other psychotic disorders, or severe affective disorders).

### Randomization and Masking

Participants who met the inclusion criteria were randomly assigned to 1 of the 4 conditions as well as to whether they would start the ambulatory smartphone-based placebo exposure by either green color or mock sound, resulting in 8 groups (1:1:1:1:1:1:1:1). Randomization was stratified by sex and included a randomly permuted block procedure with fixed block sizes of 8, 16, and 24 participants. To account for the presumably higher percentage of female participants, the female strata included 6 blocks grouped into 2 pairs of 3 blocks, each containing 1 block for each of the 3 block sizes, whereas the male strata included only 3 blocks, 1 block for each block size. Randomization was performed in RStudio (version 0.99.891; R Project for Statistical Computing [32]) by an independent party.

Participants were enrolled and assigned to the different conditions by 2 masters-level students, according to predefined rules of a standard operating procedure, which included the utilization of predefined impersonal standard emails and SMS to invite and remind participants to participate in the study. In cases of unexpected events, the master’s students communicated via email with participants, which was reduced to a necessary extent. Eligible participants were blinded to their allocation. At the end of intervention day 20, we debriefed participants via the *ohmage* app on the actual aims of the study and that we had exposed them to a placebo intervention.

### Statistical Analyses

We estimated the sample size using a priori power analysis. As no comparable RCTs were available in the literature, our assumptions regarding effect sizes were speculative. We assumed that a sample size of 30 participants in each condition (120 participants in total) would be required to detect small to moderate effects on a two-sided 5% level of significance and a power of 80%. As we anticipated an exclusion rate of 10%, we intended to assess at least 132 students for eligibility.

For descriptive analyses of baseline characteristics, we calculated absolute frequencies for categorical variables as well as means and SDs and ranges for continuous variables, each separated by condition as well as for the total number of participants. For descriptive analyses of the CEQ as an outcome measure, we first inspected histograms and Q-Q-plots for normality. As visual inspection delivered ambiguous results, we conducted the Shapiro-Wilk test, which was significant for credibility (*P*<.001) and expectancy (*P*<.001), indicating not normally distributed data. Thus, we calculated medians and IQRs.

For our main analyses, we applied linear mixed models, taking into account individual variations in efficacy expectancies across days and accommodating missing data. For the calculation of the 2 subscales credibility and expectancy of the CEQ, we equalized the 6 items of the CEQ to values from 1 to 9 according to the study by Smeets et al [31] and then calculated the row sums for each subscale. We split the 4 different conditions into 2 factors consisting of 2 levels each: prospective expectancy condition (yes vs no) and retrospective expectancy condition (yes vs no), and we entered these variables separately into the models. The variable intervention day was logarithmized with base 10 and centered. The 2 subscales of the CEQ were entered as outcome variables in separate linear mixed-effects models [33] to estimate changes in credibility or expectancy across intervention days, depending on the condition. For our main analyses, we entered the following predictors into the models: (1) intervention day (dimensional, days 0, 1, 7, 14, 20, logarithmized with base 10 and centered); (2) prospective expectancy (yes vs no); and (3) retrospective expectancy (yes vs no), as well as the interactions of intervention day with prospective expectancy and retrospective expectancy. We entered random intercept and random slope parameters as this improved model fit, the latter assessed based on Akaike Information Criterion [33], allowing time trajectories of participants to vary per participant and intervention day. With respect to our hypothesis, we were especially interested in a condition×time interaction effect (three-way interaction as condition was entered as 2 separate variables). We checked residual plots for linearity and the normal distribution of residuals.

In additional analyses, we conducted separate linear mixed models, each controlling for the effects of either prospective expectancy or retrospective expectancy. When controlling for prospective expectancy, we conducted separate models for cases with prospective expectancy (yes), respectively, without (no), and the predictors (1) intervention day and (2) retrospective expectancy, as well as the interaction of intervention day with retrospective expectancy. Likewise, when controlling for retrospective expectancy, we conducted separate models for cases with retrospective expectancy (yes), respectively, without (no), and the predictors (1) intervention day and (2) prospective expectancy, as well as the interaction of intervention day with prospective expectancy.

**Figure 2:**
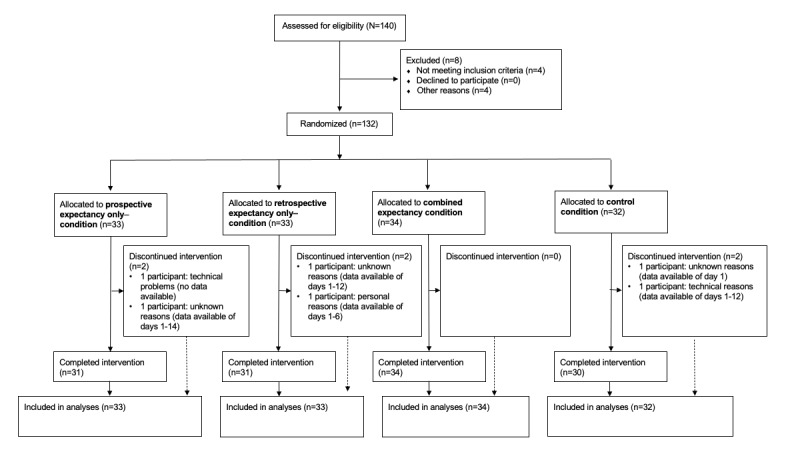
Flow of study participants. Note: as part of our intention-to-treat analyses, we included all study participants in our main analyses who were randomized to one of the 4 conditions.

We calculated 95% CIs using the Wald method. For our mixed model analyses, we included all subjects of the intention-to-treat population (Figure 2).

We conducted all tests 2-tailed and set the level of significance at .05. We used the statistical software package RStudio (version 0.99.891; R Project for Statistical Computing [32]) for all data analyses and statistical testing, including the package to conduct the mixed models *lme4* [34]. For data preparation and descriptive statistics, we used the packages *haven* [35], *dplyr* [36], *tidyr* [37], *car* [38], *ggplot2* [39], *lsmeans* [34], *lmerTest* [40], and *data.table* [41].

## Results

### Participant Flow

The flow of participants is presented in Figure 2, according to the Consolidated Standards of Reporting Trials [42]. Of the 140 participants who were assessed for eligibility, 8 were excluded before randomization because they did not meet inclusion criteria or for other reasons (eg, technical problems with their Android-based smartphones). In total, we included 132 participants in our intention-to-treat analyses.

### Baseline Characteristics of Study Participants

Baseline characteristics of study participants are presented in Table 1.

**Table 1:** Baseline characteristics of participants included in the analyses.

Variable and category			Condition					Total (N=131^a^)
			Combined^b^ (n=34)	Prospective^c^ (n=32)	Retrospective^d^ (n=33)	Control^e^ (n=32)		
**Categorical variables, n (%^f^)**								
	**Sex**							
		Male	4 (12)	5 (16)	5 (15)	5 (16)	19 (14.5)	
		Female	30 (88)	27 (84)	28 (85)	27 (84)	112 (85.5)	
	**Age group (years)**							
		18-24	32 (94)	28 (88)	26 (79)	27 (84)	113 (86.3)	
		25-29	0 (0)	2 (6)	2 (6)	3 (9)	7 (5.3)	
		30-39	1 (3)	2 (6)	3 (9)	2 (6)	8 (6.1)	
		40 or older	1 (3)	0 (0)	2 (6)	0 (0)	3 (2.3)	
	**Nationality**							
		Swiss only	23 (68)	22 (69)	22 (67)	27 (84)	94 (71.8)	
		European country	7 (21)	3 (9)	6 (18)	2 (6)	18 (13.7)	
		Other foreign country	0 (0)	0 (0)	1 (3)	0 (0)	1 (0.8)	
		Double nationality (either of them is Swiss)	4 (12)	7 (22)	4 (12)	3 (9)	18 (13.7)	
	**Education category^g^**							
		Vocational education	0 (0)	0 (0)	1 (3)	0 (0)	1 (0.8)	
		High school diploma^h^	31 (91)	30 (94)	28 (85)	30 (94)	119 (90.8)	
		University or college degree	3 (9)	2 (6)	3 (9)	2 (6)	10 (7.6)	
		Other	0 (0)	0 (0)	1 (3)	0 (0)	1 (0.8)	
	**Working part-time**							
		Yes	19 (56)	15 (47)	15 (46)	19 (59)	68 (51.9)	
		No	15 (44)	17 (53)	18 (55)	13 (41)	63 (48.1)	
	**Relationship status**							
		Single	15 (44)	16 (50)	14 (42)	14 (44)	59 (45.0)	
		In a romantic relationship	18 (53)	15 (47)	15 (46)	18 (56)	66 (50.4)	
		Married	1 (3)	0 (0)	3 (9)	0 (0)	4 (3.1)	
		Divorced	0 (0)	1 (3)	1 (3)	0 (0)	2 (1.5)	
	Occurrence of depressive symptoms for the last 2 weeks^i^		9 (27)	8 (25)	4 (12)	9 (28)	30 (22.9)	
	Occurrence of anxiousness, nervousness, strain, or excessive worriedness for the last 4 weeks^j^		0 (0)	1 (3)	0 (0)	1 (3)	2 (1.5)	
**Continuous variables, mean (SD), range (min^k^-max^l^)**								
	Full time education (years)		13.7 (2.3), (5-19)	13.7 (2.5), (5-22)	14.0 (2.4), (9-20)	13.7 (2.8), (3-20)	13.8 (2.5), (3-22)	
	Duration to complete the 20 days intervention		23.5 (3.5), (19-41)	25.1 (8.8), (19-62)	26.1 (7.2), (19-50)	23.9 (5.9), (19-62)	24.6 (6.6), (19-62)	
	PSS-10^m^		16.1 (4.8), (6-26)	15.4 (4.8), (5-25)	15.2 (6.6), (4-30)	17.0 (6.9), (4-32)	15.9 (5.8), (4-32)	

^a^Although we included all 132 study participants of the intention-to-treat sample in our main analyses, here, we report the data of only 131 study participants because for 1 study participant, the data presented here were missing.

^b^Combined: combined expectancy condition.

^c^Prospective: prospective expectancy–only condition.

^d^Retrospective: retrospective expectancy–only condition.

^e^Control: control condition.

^f^Percentages may not total 100% due to rounding.

^g^The categories no formal education, compulsory education, and higher vocational education (including school for technicians and professional school) have been dropped due to the lack of cases.

^h^In the German version of the questionnaire, participants were asked for *Matura*, which is the secondary school leaving certificate in Switzerland, equivalent to International Standard Classification for Education 34.

^i^Items of the Patient Health Questionnaire-German version, with the following scale: 1=not at all, 2=on single days, 3=on more than half of the days, and 4=almost every day, categorization according to meeting at least the criteria for other depressive syndrome according to the manual of the Patient Health Questionnaire-German version.

^j^Items of Patient Health Questionnaire-German version, with the following scale: 1=no and 2=yes, categorization according to meeting at least the criteria for panic syndrome or other anxiety syndromes.

^k^min: minimum.

^l^max: maximum.

^m^PSS-10: Perceived Stress Scale, 10 items version.

### Results From the Mixed Model Analyses

#### Credibility

**Figure 3:**
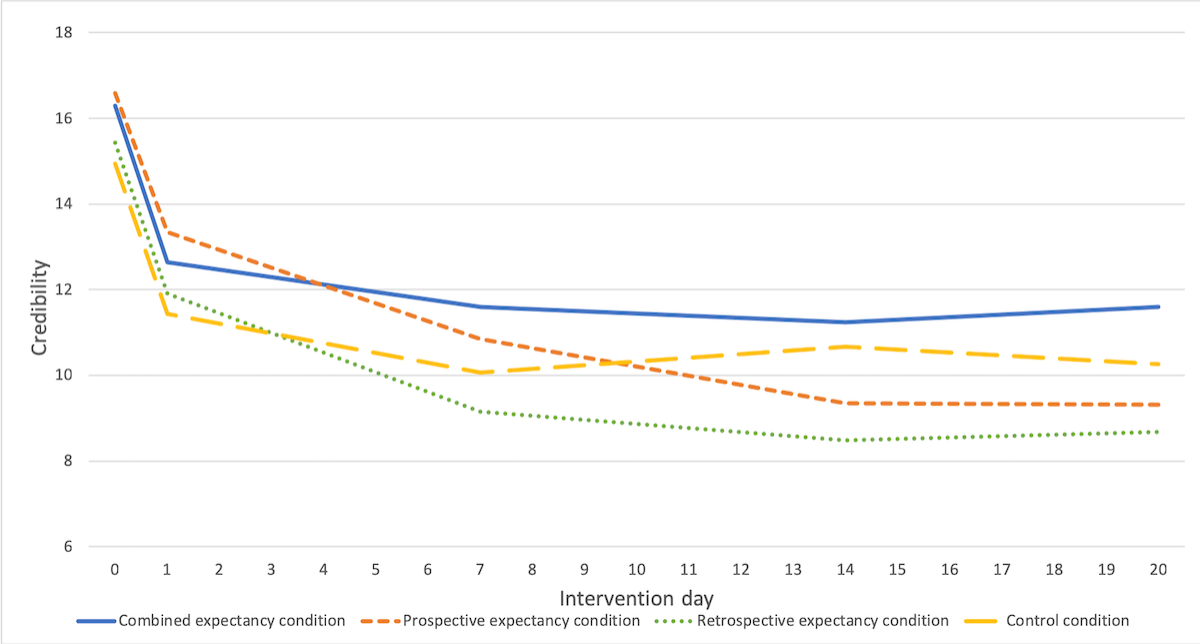
Time trajectories of credibility throughout intervention days (means). Note: means were calculated based on existing values.

Descriptive statistics are shown in Table 2 and in the interaction plots of Figure 3. The results of the main mixed models are presented in Table 3. We found a significant main effect of *intervention day* (β=−1.63; 95% CI −2.37 to −0.89; *P*<.001), suggesting that credibility decreased over the intervention days, irrespective of condition. We found a significant three-way interaction: intervention day×prospective expectancy×retrospective expectancy (β=2.05; 95% CI 0.60-3.50; *P*=.006). Results from additional analyses (Multimedia Appendix 4) suggest that the significant three-way interaction was driven by 2 opposite two-way interactions: intervention day×retrospective expectancy, one positive in cases with prospective expectancy (β=1.18; 95% CI 0.31-2.05; *P*=.009) and one negative in cases with no prospective expectancy (β=−0.87; 95% CI −2.06 to 0.33; *P*=.15), with 95% CIs of estimates minimally overlapping. When controlling for retrospective expectancy, the two-way interaction pattern intervention day×prospective expectancy was comparable. In line with Figure 3, these findings suggest that credibility decreased least in the combined expectancy condition and in the control condition.

**Table 2:** Outcome measures: median scores of the Credibility and Expectancy Questionnaire per intervention day.

Outcome and intervention day		Condition								Total (N=131)^a^	
		Combined^b^ (n=34)		Prospective^c^ (n=33)		Retrospective^d^ (n=33)		Control^e^ (n=32)			
		Median (IQR)	Number of patients	Median (IQR)	Number of patients	Median (IQR)	Number of patients	Median (IQR)	Number of patients	Median (IQR)	Number of patients
**Credibility**											
	0	16 (4.75)	34	16 (5.25)	32	16 (4)	33	15 (4.25)	32	16 (4)	131
	1	12.5 (7)	34	14 (5.5)	32	12 (5)	33	12.5 (7.25)	32	13 (7)	131
	7	11.5 (7)	34	11 (8)	32	8 (7)	32	10 (8)	31	10 (8)	129
	14	12.5 (8)	34	9.5 (6.75)	32	8 (6)	31	10 (6.75)	30	9 (8.5)	127
	20	11.5 (6.5)	32	8 (7)	31	7 (7.5)	31	9.5 (8.75)	30	9 (9)	124
**Expectancy**											
	0	11.5 (5.75)	34	12 (4.5)	32	11 (5)	33	10 (7.75)	32	11 (5.25)	131
	1	10.5 (4.75)	34	10 (5.25)	32	9 (4)	33	11 (7.25)	32	10 (6)	131
	7	10 (5)	34	8 (5.25)	32	6 (3.25)	32	8 (5.5)	31	8 (5)	129
	14	8.5 (5)	34	7 (5.5)	32	6 (5.5)	31	7.5 (10)	30	7 (7)	127
	20	7.5 (5.25)	32	7 (3.5)	31	6 (4.5)	31	8.5 (9.75)	30	7 (7)	124

^a^Although we included all 132 study participants of the intention-to-treat sample in our main analyses, here, we report the data of only 131 study participants because for 1 study participant, the data presented here were missing. Means and SDs were calculated based on the existing values. N values per intervention day and condition are given in the table.

^b^Combined: combined expectancy condition.

^c^Prospective: prospective expectancy–only condition.

^d^Retrospective: retrospective expectancy–only condition.

^e^Control: control condition.

**Table 3:** Results of linear mixed models (N=131)^a^.

Predictors^b^			*b*		95% CI		*P* values
**Credibility**							
	Intercept	11.35		10.04 to 12.66		<.001	
	Intervention day (time^c^; logarithmized)	−1.630		−2.37 to −0.89		<.001	
	PE^d^	0.497		−1.35 to 2.34		.60	
	RE^e^	−0.590		−2.43 to 1.25		.53	
	Time×PE	−1.130		−2.16 to −0.09		.03	
	Time×RE	−0.869		−1.91 to 0.17		.10	
	PE×RE	1.467		−1.11 to 4.05		.26	
	Time×PE×RE	2.046		0.60 to 3.50		.006	
**Goodness of fit**							
	AIC^f^	3559.2		—^g^		—	
**Expectancy**							
	Intercept	9.715		8.45 to 10.98		<.001	
	Intervention day (time; logarithmized)	−0.770		−1.49 to −0.05		.04	
	PE	−0.425		−2.21 to 1.36		.64	
	RE	−1.373		−3.15 to 0.40		.13	
	Time×PE	−0.871		−1.88 to 0.13		.09	
	Time×RE	−0.744		−1.75 to 0.26		.15	
	PE×RE	2.099		−0.39 to 4.59		.10	
	Time×PE×RE	1.548		0.14 to 2.95		.03	
**Goodness of fit**							
	AIC	3378.7		—		—	

^a^We included 132 study participants of the intention-to-treat sample in our data set. As from 1 participant there were no data available for at least one intervention day, statistical analyses were conducted with the data of only 131 participants.

^b^For interpretation purposes, we entered the 4 conditions as 2 separate variables: prospective expectancy (PE; yes vs no) and retrospective expectancy (RE; yes vs no) in the mixed models. This means combined expectancy condition corresponds to PE=yes and RE=yes, prospective expectancy–only condition corresponds to PE=yes and RE=no, retrospective expectancy–only condition corresponds to PE=no and RE=yes, and control condition corresponds to PE=no and RE=no.

^c^time: intervention day.

^d^PE: prospective expectancy (yes vs no).

^e^RE: retrospective expectancy (yes vs no).

^f^AIC: Akaike information criterion.

^g^—: not available.

#### Expectancy

**Figure 4:**
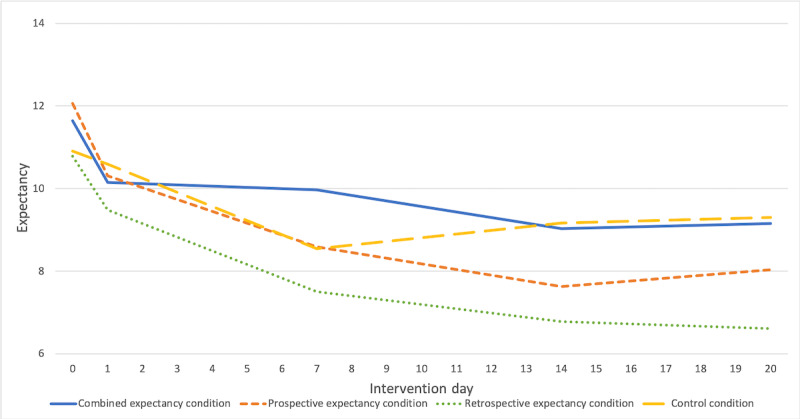
Time trajectories of expectancy throughout intervention days (means). Note: means were calculated based on existing values.

Descriptive statistics can be found in Table 2 and in the interaction plots of Figure 4. The results of the main mixed models are presented in Table 3. We found a significant main effect of *intervention day* (β=−0.77; 95% CI −1.49 to −0.05; *P*=.04), suggesting that expectancy decreased over the intervention days. We found a significant three-way interaction: intervention day×prospective expectancy×retrospective expectancy (β=1.55; 95% CI 0.14-2.95; *P*=.03). Results from additional analyses (Multimedia Appendix 5) suggest that the significant three-way interaction was driven by 2 opposite two-way interactions: intervention day×retrospective expectancy, one positive in cases with prospective expectancy (β=.81; 95% CI −0.01 to 1.62; *P*=.05) and one negative in cases with no prospective expectancy (β=−.74; 95% CI −1.92 to 0.44; *P*=.21), with 95% CIs of estimates minimally overlapping. When controlling for *retrospective expectancy*, the two-way interaction pattern intervention day×prospective expectancy was comparable. In line with Figure 4, these findings suggest that expectancy decreased least in the combined expectancy condition and in the control condition.

## Discussion

### Principal Findings

To the best of our knowledge, this is the first empirical study investigating whether efficacy expectancies can be successfully induced in a smartphone-based placebo mental health intervention. We found that efficacy expectancies decreased throughout the intervention days, irrespective of the condition. Efficacy expectancies decreased least in the combined expectancy and the control condition and most in the prospective expectancy–only condition and the retrospective expectancy–only condition.

The finding that efficacy expectancies decreased throughout intervention days may partly be explained by the length and monotony of the intervention. Some participants mentioned in their feedback at the end of the intervention that the duration of the smartphone-based intervention and the daily exposure to green color or mock sound were too long.

Efficacy expectancies decreased least in the combined expectancy and in the control condition, followed by the prospective expectancy–only and the retrospective expectancy–only condition. As displayed in Figures 3 and 4, the verbal instructions given in the prospective expectancy–only condition alone did not seem to have an effect on efficacy expectancies, as they continued to decrease after intervention day 1, on which the instructions had been given. A potential explanation may be that our verbal instructions were not potent enough to raise efficacy expectancies. Accordingly, Rief et al [43] encouraged study participants of the PSY-HEART study to develop very clear expectations of how their daily life would change after successful heart surgery. Due to these more personal associations, study participants may have been more convinced and may have formed stronger expectancies. Future studies may further explore potential study protocols to maximize expectancies, regarding types (eg, conditioning procedure instead of verbal instructions [14,16]), timepoint, number of repetitions, and intervals of expectancy induction.

### Strengths and Limitations

Our study has several strengths. First, we set up the study in the frame of an RCT, which is the gold standard in psychotherapy research. Second, the smartphone-based placebo intervention as well as efficacy expectancies were delivered in a standardized way by providing them in the preprogrammed surveys in the Android-based smartphone app as well as on the web-based platform, through which heterogeneity due to different experimenters or protocols could be reduced. Although randomization and allocation concealment were not done automatically in the Android-based smartphone app but by experimenters, in most cases, they did not have any personal contact with study participants after randomization, thereby reducing experimenter bias. Third, our results have high ecological validity, because participants used their own smartphones in daily life at a specific time of the day and from intervention day 15 in situations when they felt stressed. Fourth, by adapting the open-source *ohmage* app to the purpose of our study, we provide a minimal cost intervention that enables fully identical replications as well as their utilization in low- and middle-income countries in which there is a lack of financial resources. Fifth, with the use of mixed model analyses, we took into account individual variations in efficacy expectations across intervention days and accounted for missing data.

Our results may be interpreted in light of several limitations. First, we designed the smartphone-based intervention in line with the aim of our study to create an inert intervention. We did not focus on making the intervention particularly attractive to study participants, for instance, by integrating elements of gamification [44], which may have affected the decrease in efficacy expectancies throughout the intervention. It may be hypothesized that the time trajectories in efficacy expectancies may differ in a study using a smartphone-based app designed to have a specific effect. Still, in contrast to the law of attrition, which describes the observation of high rates of discontinuation in eHealth trials [45], we have a high completion rate, with data available for 97.3% (642/660) of cases, which makes our findings relatively robust. Second, the study sample was quite homogenous, with most of the participants being female psychology students. As our sample did not consist of a clinical sample, it may rather reflect subjects using smartphones for preventive purposes. Hence, the findings may be generalizable rather to populations seeking prevention. Notably, in a clinical population, participants’ desire to get an effect out of the intervention is expected to be higher than in a healthy sample, which has been found to modulate placebo analgesia in irritable bowel syndrome patients [46]. Therefore, it may even be easier to induce a digital placebo effect in a clinical sample, as compared with ours, which, however, requires further investigation. Another limitation regarding our sample is that data collection took place in 2015, and it would have been preferable to use more recent data, particularly in a fast-emerging field such as mobile health. However, the latency between data collection and dissemination of findings in our study is comparable with other relevant studies in this field [23,47]. Furthermore, although timely dissemination of findings from clinical trials would be important to base clinical decisions on best scientific evidence, a previous study [48] found that only 29% of completed RCTs of US academic medical centers are published within 2 years after study completion, indicating that, to reduce publication bias, older data need to be disseminated as well. Nonetheless, our findings require imminent replication. For replication, it would also be preferable to increase the sample size, which, however, encompassed 132 participants in our main statistical analyses; thus, it was above the median of comparable RCTs included in 2 recent meta-analyses [11,12]. Third, some participants reported technical or usability problems with the *ohmage* app, which may have led to a certain level of frustration throughout the course of the intervention and may have diminished efficacy expectancies. However, as the reported frequency of technical problems with the app was low (0.6% of all cases), we do not assume that this aspect has reduced the validity of our findings. Fourth, as participants entered the study at different points of time, we cannot exclude that participants who had already finished the study might have informed others about the actual study purpose before study completion, which may have reduced the effect of the induction of efficacy expectations, particularly in the experimental conditions. However, we speculate that this may have affected efficacy expectancies of only a few participants because (1) participants might not have remembered and passed all the details of the study design to others; (2) it might have been in the interest of most of the psychology students to promote the study; and (3) students from fields other than psychology might have not systematically participated, and thus, they might not have shared details of the purpose of the study. Fifth, we included only participants with access to an Android-based smartphone, which limits the generalizability to iPhone and other operating systems users. However, a recent study found that personality traits (eg, well-being, self-esteem, optimism, pessimism, and the Big Five) that might affect efficacy expectancies differed only slightly between iOS and Android users [49].

### Implications

In this study, we focused on the investigation of whether we succeeded in inducing efficacy expectancies in a smartphone-based placebo mental health intervention. A required next step would be to investigate whether the induction of efficacy expectancies affected behavioral outcomes, such as mood and stress (Stalujanis et al, unpublished data, January 2021). In the field of placebo research, there are situations in which placebo effects should be diminished and others in which placebo effects should be enhanced [15,21]. Further investigation of time trajectories of efficacy expectancies as a potential mechanism of digital placebo effects may help to improve research on the efficacy of smartphone-based mental health interventions by disentangling digital placebo effects from specific effects. A potential study design may provide participants with a smartphone-based inert intervention until placebo effects are supposed to be flattened and then deploy the actual intervention, which may then be less distorted by placebo effects. In addition, it is well known that, after initial involvement, users of digital mental health interventions tend to put those away [50]. If smartphone-based mental health interventions work only every second or third time, users may lose their motivation and might not see any gain from the intervention. Personalized prediction of the effects of efficacy expectancies may foster long-term utilization of smartphone-based interventions. In a previous study, we found that, by using a machine learning approach, predictions of smartphone-based psychotherapeutic microintervention success could be improved, as compared with the initial success rate within and between participants [51]. Future studies should investigate predictors of efficacy expectancies at an intra- and interindividual level to design tailor-made individualized interventions to contribute to further advancement in the growing field of precision medicine [52].

### Conclusions

To the best of our knowledge, this is the first empirical study investigating whether efficacy expectancies could be successfully induced in a smartphone-based placebo mental health intervention. We found that efficacy expectancies decreased throughout the intervention days. Efficacy expectancies decreased least in the combined expectancy condition and in the control condition and most in the retrospective expectancy–only condition and the prospective expectancy–only condition. Our findings may pave the way for both diminishing and exploiting effects of outcome expectancies as a potential mechanism of the digital placebo effect and help to improve the treatment efficacy of digital mental health interventions.

## Appendix

Multimedia Appendix 1 Outline of the larger study.

Multimedia Appendix 2 Study design of the larger study.

Multimedia Appendix 3 Instructions of the four conditions of the study. Note. Those instructions were translated from German to English by the first author for illustration purposes. The original German original versions are available on request by the authors.

Multimedia Appendix 4 Results of additional analyses of linear mixed models with credibility as outcome (N=131).

Multimedia Appendix 5 Results of additional analyses of linear mixed models with expectancy as outcome (N=131).

Multimedia Appendix 6 CONSORT-eHEALTH checklist (V 1.6.1).

## Abbreviations

CEQ: Credibility and Expectancy Questionnaire
RCT: randomized controlled trial
