# Supplementary material for: Induction of Efficacy Expectancies in an Ambulatory Smartphone-Based Digital Placebo Mental Health Intervention: Randomized Controlled Trial
Source: JMIR Mhealth Uhealth. 2021 Feb 17;9(2):e20329. doi: 10.2196/20329 (PMC7929742; doi:10.2196/20329)
Supplement: Multimedia Appendix 1 [file mhealth_v9i2e20329_app1.pdf]

Multimedia Appendix 1. Outline of the larger study

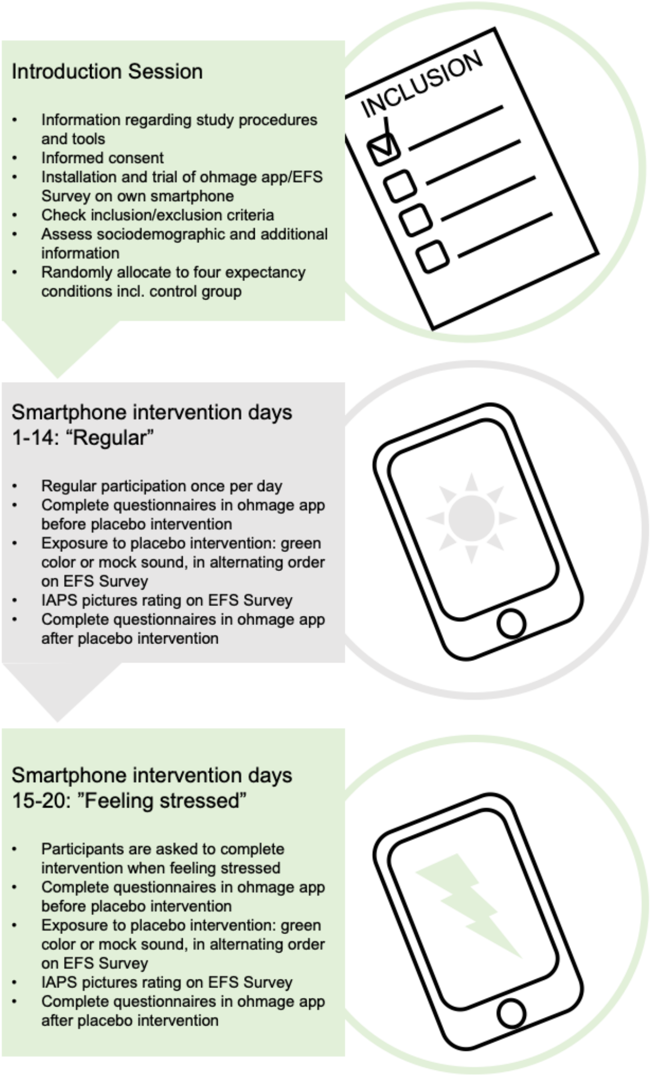

Abbreviations: IAPS - International Affective Picture System
