# Supplementary material for: Induction of Efficacy Expectancies in an Ambulatory Smartphone-Based Digital Placebo Mental Health Intervention: Randomized Controlled Trial
Source: JMIR Mhealth Uhealth. 2021 Feb 17;9(2):e20329. doi: 10.2196/20329 (PMC7929742; doi:10.2196/20329)
Supplement: Multimedia Appendix 2 [file mhealth_v9i2e20329_app2.pdf]

Multimedia Appendix 2: Study design of the larger study

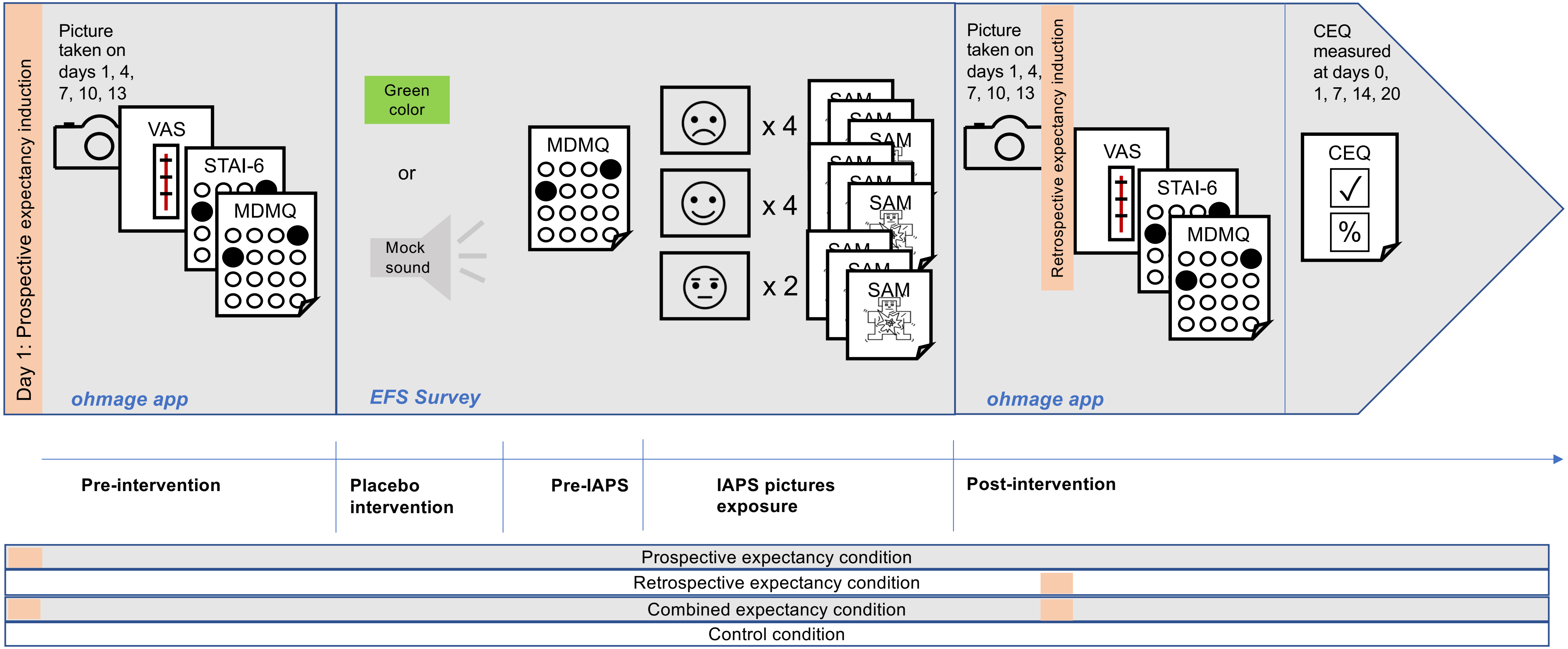

Abbreviations: CEQ – Credibility Expectancy Questionnaire; IAPS – International Affective Picture System; MDMQ – Multidimensional Mood State Questionnaire; SAM – Self-Assessment Manikin; STAI-6: short form of the Spielberger State-Trait Anxiety Inventory; VAS – visual analog scale
