# Supplementary material for: Induction of Efficacy Expectancies in an Ambulatory Smartphone-Based Digital Placebo Mental Health Intervention: Randomized Controlled Trial
Source: JMIR Mhealth Uhealth. 2021 Feb 17;9(2):e20329. doi: 10.2196/20329 (PMC7929742; doi:10.2196/20329)
Supplement: Multimedia Appendix 3 [file mhealth_v9i2e20329_app3.pdf]

Multimedia Appendix 3. Instructions of the four conditions of the study

Condition	Instruction
Control	General introduction:<br>*"Thank you for participating in our smartphone study. You help us to find out how mood and perceived stress fluctuate in daily life and whether smartphones are suitable to capture their trajectories."*<br><br>Intervention day 1:<br>*"Welcome to our smartphone study. We are interested how mood and perceived stress vary in daily life and whether smartphones are suitable to capture their trajectories. During the next three weeks you will complete questionnaires on stress and your mental state and rate affective pictures on a daily basis. Additionally, we will ask you to watch a green picture during two minutes on each day or to open yourself to a non-audible tone on your smartphone. The tone is a very gentle sound, which is not audible for the human ear, and does not pose a danger to your health."*<br><br>*"On some days, we will additionally ask you to take a self-portrait with your smartphone, before and after the rating of emotional pictures."*<br><br>Intervention days 1, 4, 7, 10, 13 (pre-intervention):<br>*"Please take a self-portrait with your smartphone camera on the following site by ticking the box with the camera symbol. Your smartphone camera*

will be turned on automatically. Please be aware that your whole face should be on the picture and try to avoid sudden movements."

"Fine! Thank you! Please click on 'Next' to continue with the next task."

<u>Intervention days 1, 4, 7, 10, 13 (post-intervention):</u>

"Please again take a self-portrait with your smartphone camera on the following site by ticking the box with the camera symbol. Your smartphone camera will be turned on automatically. Please be aware that your whole face should be on the picture and try to avoid sudden movements."

<u>Intervention days 1, 4, 7, 10, 13 (post-intervention):</u>

<u>feedback on self-portrait:</u>

"Thank you very much!"

Prospective expectancy

<u>General introduction:</u>

"Thank you for participating in our smartphone study. You help us to find out whether daily exposure to green light and a soft tone in the course of a smartphone intervention lasting several weeks may have a positive effect on mood and stress perception."

<u>Intervention day 1:</u>

"Welcome to our smartphone study. We are interested in the effects of a smartphone intervention lasting several weeks on mood and stress perception. During the next three weeks, you will complete questionnaires

on stress and your mental state as well as rate affective pictures on a daily basis. Additionally, we will ask you to watch a green picture during two minutes on each day or to open yourself to a non-audible tone on your smartphone. The tone is a very soft sound, which is not audible for the human ear, and does not pose a danger to your health. Previous studies found that green light and soft tones beyond perception threshold may positively affect the activity of certain brain regions, such as the so-called insula. The insula is involved in the formation of unpleasant emotions (e.g. anger, fear, sadness, disgust) and the release of stress hormones, such as cortisol. Therefore, we assume that the daily exposure to a green picture or soft tone will positively affect your mood as well as your stress perception in general and particularly at the rating of emotional pictures."

"On some days, we will additionally ask you to take a self-portrait with your smartphone, before and after the rating of emotional pictures."

<u>Intervention days 1, 4, 7, 10, 13 (pre-intervention):</u>

Instructions identical to the control condition.

<u>Intervention days 1, 4, 7, 10, 13 (post-intervention):</u>

Instructions identical to the control condition.

<u>Intervention days 1, 4, 7, 10, 13 (post-intervention):</u>

<u>feedback on self-portrait:</u>

Instructions identical to the control condition.

Retrospective expectancy	General introduction:
	Instructions identical to the control condition.

Intervention day 1:

Instructions identical to the control condition, except:

"On some days, we will additionally ask you to take a self-portrait with your smartphone, before and after the rating of emotional pictures. The ohmage app will compare your self-portraits regarding your emotional facial expression, which may vary according to mood and perceived stress. Subsequently, you will receive a short feedback on picture analysis."

Intervention days 1, 4, 7, 10, 13 (pre-intervention):

Instructions identical to the control condition.

Intervention days 1, 4, 7, 10, 13 (post-intervention):

Instructions identical to the control condition, except for:

"Your picture is currently being analyzed. Please click on 'Next' now."

Intervention days 1, 4, 7, 10, 13 (post-intervention):

feedback on self-portrait:

Intervention day 1:

"Your stress level and your mood improved about 20 per cent."

Intervention day 4:

"Your stress level and your mood improved about 30 per cent."

	Intervention day 7:<br>*"Your stress level and your mood improved about 50 per cent."*<br>Intervention day 10:<br>*"Your stress level and your mood improved about 40 per cent."*<br>Intervention day 13:<br>*"Your stress level and your mood improved about 60 per cent."*
Combined expectancy	General introduction:<br>*Instructions identical to the prospective expectancy condition.*<br><br>Intervention day 1:<br>*"Welcome to our smartphone study. We are interested in the effects of smartphone intervention lasting several weeks on mood and stress perception. During the next three weeks you will complete questionnaires on stress and your mental state and rate affective pictures on a daily basis. Additionally, we will ask you to watch a green picture during two minutes on each day or to open yourself to a non-audible ton on your smartphone. The ton is a very gentle sound, which is not audible for the human ear, and does not pose a danger to your health. Previous studies found that green light and soft tons beyond perception threshold may positively affect the activity of certain brain regions, such as the so-called insula. The insula is involved in the formation of unpleasant emotions (e.g. anger, anxiety, grief, disgust) and the release of stress hormones, such as cortisol. Therefore, we assume that daily exposure to green picture or soft ton will*

positively affect your mood as well as your stress perception in general and particularly at the rating of emotional pictures."

"On some days, we will additionally ask you to take a self-portrait with your smartphone, before and after the rating of emotional pictures. The ohmage app will compare your self-portraits regarding your emotional facial expression, which may vary according to mood and perceived stress. Subsequently, you will receive a short feedback on picture analysis."

<u>Intervention days 1, 4, 7, 10, 13 (pre-intervention):</u>

Instructions identical to the control condition.

<u>Intervention days 1, 4, 7, 10, 13 (post-intervention):</u>

Instructions identical to the control condition.

<u>Intervention days 1, 4, 7, 10, 13 (post-intervention): feedback on self-portrait:</u>

Instructions identical to the retrospective expectancy condition.

Note. The above-listed instructions have been translated from German to English by the first author for illustration purposes. German original versions are available on request by the authors.
