# Supplementary material for: Induction of Efficacy Expectancies in an Ambulatory Smartphone-Based Digital Placebo Mental Health Intervention: Randomized Controlled Trial
Source: JMIR Mhealth Uhealth. 2021 Feb 17;9(2):e20329. doi: 10.2196/20329 (PMC7929742; doi:10.2196/20329)
Supplement: Multimedia Appendix 4 [file mhealth_v9i2e20329_app4.docx]

| Multimedia Appendix 4. Results of additional analyses of linear mixed models with credibility as outcome (*N*=131)^a^. | | | | | | | | | | | |
| --- | --- | --- | --- | --- | --- | --- | --- | --- | --- | --- | --- |
|  | Model 1: PE = no (*n*=65, *n obs.*=315) | | | | | Model 2: PE = yes (*n*=66, *n obs.*=327) | | | | | |
| Predictors^b^ | *b* | 95% CI | | *P* |  | *b* | 95% CI | | *P* | |  |
| (Intercept) | 11.33 | 10.06; | 12.61 | < .001 | *** | 11.85 | 10.48; | 13.21 | < .001 | *** | |
| Intervention day (time; log.) | -1.64 | -2.50; | -0.79 | < .001 | *** | -2.76 | -3.39; | -2.13 | < .001 | *** | |
| Prospective Expectancy (PE) | = 0 | | | | | = 1 | | | | | |
| Retrospective Expectancy (RE) | -0.59 | -2.37; | 1.20 | .51 |  | 0.88 | -1.02; | 2.78 | .36 |  | |
| Time*RE | -0.87 | -2.06; | 0.33 | .15 |  | 1.18 | 0.31; | 2.05 | .009 | ** | |
| Goodness-of-fit | | | | | | | | | | | |
| AIC | 1765.9 | | | | | 1793.3 | | | | | |
|  | | | | | | | | | | | |
|  | Model 3: RE = no (*n*=64, *n obs.*=314) | | | | | Model 4: RE = yes (*n*=67, *n obs.*=328) | | | | | |
| Predictors^a^ | *b* | 95% CI | | *P* |  | *b* | 95% CI | | *P* | |  |
| (Intercept) | 11.34 | 9.96; | 12.72 | < .001 | *** | 10.77 | 9.52; | 12.02 | < .001 | *** | |
| Intervention day (time; log.) | -1.64 | -2.46; | -0.82 | <. 001 | *** | -2.49 | -3.16; | -1.82 | < .001 | *** | |
| Prospective Expectancy (PE) | 0.51 | -1.44; | 2.45 | .60 |  | 1.96 | 0.21; | 3.70 | .03 | * | |
| Retrospective Expectancy (RE) | = 0 | | | | | = 1 | | | | | |
| Time*PE | -1.12 | -2.26; | 0.03 | .06 |  | 0.91 | -0.02; | 1.84 | .06 |  | |
| AIC | 1761.7 | | | | | 1802.9 | | | | | |

Abbreviations: AIC – Akaike information criterion; *n obs.* – number of observations; PE – prospective expectancy (yes vs. no); RE – retrospective expectancy (yes vs. no); time – intervention day

^a^We included 132 study participants of the intention-to-treat sample in our dataset. As from one participant there was no data available for at least one intervention day, statistical analyses were conducted with the data of only 131 participants.

^b^For interpretation purpose, we entered the four conditions as two separate variables ‘prospective expectancy’ (PE; yes vs. no) and ‘retrospective expectancy’ (RE; yes vs. no) in the mixed models.
